# Multisite harmonization of diffusion tensor image analysis along the perivascular space using the COMBined Association Test

**DOI:** 10.1007/s11604-023-01432-z

**Published:** 2023-04-24

**Authors:** Yuya Saito, Koji Kamagata, Christina Andica, Toshiaki Taoka, Rukeye Tuerxun, Wataru Uchida, Kaito Takabayashi, Mana Owaki, Seina Yoshida, Keigo Yamazaki, Shinji Naganawa, Shigeki Aoki

**Affiliations:** 1 Department of Radiology, Juntendo University Graduate School of Medicine, 2-1-1 Hongo, Bunkyoku, Tokyo, 113-8421 Japan; 2 Faculty of Health Data Science, Juntendo University, Chiba, Japan; 3 Department of Innovative Biomedical Visualization (iBMV), Nagoya University Graduate School of Medicine, Nagoya, Japan; 4 Department of Radiological Sciences, Graduate School of Human Health Sciences, Tokyo Metropolitan University, Tokyo, Japan; 5 Department of Radiology, Nagoya University Graduate School of Medicine, Nagoya, Japan

**Keywords:** Multisite study, Harmonization, Diffusion magnetic resonance imaging, Diffusion tensor imaging along the perivascular space index

## Abstract

**Purpose:**

This multisite study aimed to use the COMBined Association Test (COMBAT), a harmonization technique that uses regression of covariates with an empirical Bayesian framework, to harmonize diffusion tensor image analysis along the perivascular space (DTI-ALPS) variations caused by scanner, site, and protocol differences.

**Materials and methods:**

This study included multisite diffusion magnetic resonance imaging (dMRI) data of 45 patients with Alzheimer’s disease (AD) and 82 cognitively normal (CN) participants from the AD neuroimaging initiative database. The dMRI data were obtained with two *b* values (0 and 1000 s/mm^2^) from 27 institutions and three different 3-Tesla MRI scanners (two vendors). The ALPS index was calculated from multisite dMRI data, and COMBAT was used to harmonize the factors causing site variations. Welch’s *t* test was used, Cohen’s *d* was calculated to compare the difference in the ALPS index between AD and CN before and after harmonization, and Pearson’s correlation coefficient was calculated to assess the relationships between the ALPS index and the cognitive score, [^18^F] fluorodeoxyglucose (FDG)-positron emission tomography (PET), and [^18^F] florbetapir (AV45)-PET standardized uptake value ratios (SUVRs).

**Results:**

COMBAT harmonized scanner differences and increased Cohen’s *d* of the left and right ALPS indexes between AD and CN from 0.288 to 0.438 and 0.328 to 0.480, respectively. The ALPS indexes were significantly different between AD and CN after harmonization (*P* < 0.05) but not before it. Moreover, Pearson’s correlation coefficients between the ALPS index and cognitive score, FDG-PET, and AV45-PET SUVRs were higher after harmonization than before it.

**Conclusion:**

This study demonstrates the application of COMBAT harmonization to eliminate between-scanner, site, and protocol variations in the ALPS index calculated from DTI-ALPS using dMRI and possibly facilitate the use of the ALPS index in multi-center studies.

## Introduction

The glymphatic system plays a role in the excretion of waste products, including neurotoxins such as amyloid β (Aβ) and tau protein, which are a pathological hallmark of Alzheimer’s disease (AD). Glymphatic system dysfunction in the rodent brain has been shown to suppress the clearance of soluble Aβ [1, 2]; however, the relationship between the glymphatic system and AD pathogenesis in humans remains unknown.

The glymphatic hypothesis [1, 2] states that the subarachnoid cerebrospinal fluid (CSF) enters the brain interstitial space from the periarterial space via the aquaporin-4 channel expressed in astrocyte end-feet and then mixes with the interstitial fluid (ISF) and waste solutes in the brain. Subsequently, the resulting CSF/ISF exchange and waste products, such as Aβ and tau protein, drain out of the brain via the perivenous efflux pathway. The major method used to evaluate in vivo glymphatic system activity is to conduct follow-up tracer studies on intrathecal administration of a gadolinium-based contrast agent (GBCA) in magnetic resonance imaging (MRI) that can visualize ISF dynamics in an animal model, such as a mouse [3, 4]. However, studies in human subjects are limited because intrathecal administration of GBCA for MRI is invasive for the subject. Taoka et al. [5] proposed diffusion tensor image analysis along the perivascular space (DTI-ALPS) based on diffusion MRI (dMRI) as a non-invasive method for imaging the glymphatic system without the requirement of intrathecal injection of a contrast agent to evaluate ISF dynamics [1, 2]. The DTI-ALPS method provides an ALPS index, which is a ratio of the diffusivity in the perivascular space direction and the direction of perpendicular diffusivity to both the major fiber tract and perivascular spaces [5, 6]. Recently, the DTI-ALPS method has been used to show alterations of intestinal fluid dynamics in the glymphatic system associated with various pathologies [7–14]. AD, in particular, is strongly correlated with the ALPS index; this index is lower in individuals with AD and mild cognitive impairment (MCI) than in those who are cognitively normal (CN) [5, 15].

A multisite study using the ALPS index emphasized the importance of considering the reproducibility of the ALPS index and the effect of various factors, such as MRI scanner and acquisition parameters, because the DTI parameter-based ALPS index is susceptible to variations caused by differences in the scanners, sites, and protocols used. Taoka et al. [16] revealed that the ALPS index was influenced by the number of motion-proving gradient (MPG) axes and echo time (TE) in the imaging protocol and MRI scanner. Additionally, the ALPS index was affected by scanner differences even with the same imaging protocol. Therefore, MRI scanner and acquisition parameters should be uniform to eliminate measurement bias and focus on the sampling bias, such as pathology, in participants. However, multisite population studies with large datasets include various MRI scanners and acquisition parameters; two examples are the Alzheimer’s Disease Neuroimaging Initiative (ADNI) [17] cohort of 5100 participants and the Parkinson’s Progression Markers Initiative cohort [18] of 4300 participants. These differences lead to variability, low reproducibility, and/or bias in the detection of disease-related changes [19, 20]. However, a large sample size is required to detect a pathological change with high reproducibility and reliability by increasing the statistical power and establishing evidence for the ALPS index.

In the last decade, novel approaches have been developed to statistically harmonize neuroimaging-based metrics acquired from different sites that used different scanners and/or protocols. The first attempts were to utilize sites as covariates in a generalized linear model (GLM) [21]. However, the GLM-based harmonization method could not eliminate measurement bias; thus, biological information related to neuropathology, sex, and age could still result in bias. The COMBined Association Test (COMBAT), which is an extension of GLM with an empirical Bayesian-based harmonization method, was then developed [21]. In their study, they adapted and compared several statistical approaches for the harmonization of multisite DTI metrics including fractional anisotropy (FA) and the mean diffusivity (MD) that were previously developed for other data types: functional normalization [22], RAVEL [23], surrogate variable analysis [24], and COMBAT [25], a popular batch adjustment method developed for genomics data. They also included a simple method that globally rescales the data for each site using a z-score transformation map common to all features, which they refer to as “global scaling.” Then they proposed and compared several harmonization approaches for DTI data and showed that COMBAT exhibited the best performance in terms of modeling and removing unwanted intersite variability in FA and MD maps [21]. Using age as a biological phenotype of interest, they showed that COMBAT preserved biological variability as well as removed the unwanted variation introduced by site differences [21]. However, to the best of our knowledge, no previous study has assessed the performance of COMBAT in harmonizing the ALPS index based on DTI-ALPS using a multisite dMRI dataset, including variations in scanners, sites, and protocols. For these reasons, COMBAT harmonization was performed in this study.

This study aimed to validate methods for harmonizing the ALPS index based on DTI-ALPS using data from a multisite population study on patients with AD and CN; the data were collected from the ADNI database (https://adni.loni.usc.edu). We statistically evaluated the performance of COMBAT in terms of harmonizing the ALPS index’s variations in scanners, sites, and protocols and assessed the effect of COMBAT harmonization on improving the correlations between the ALPS index and the cognitive score, [^18^F] fluorodeoxyglucose (FDG)-positron emission tomography (PET), and [^18^F] florbetapir (AV45)-PET standardized uptake value ratios (SUVRs).

## Materials and methods

### Study cohorts

Study data were obtained from the ADNI-3 database (https://adni.loni.usc.edu). ADNI was launched in 2003 as a public–private partnership, led by principal investigator Michael W. Weiner, MD, VA Medical Center and University of California, San Francisco. ADNI primarily aimed to test the combination of serial MRI, PET, other biological markers, and clinical and neuropsychological assessments to measure the progression of MCI and early AD. A more detailed and up-to-date description of the ADNI is provided at www.adni-info.org.

The inclusion and exclusion criteria for AD and CN were as follows: (1) dMRI was acquired using a series description “axial DTI,” including a three-scanner model and acquisition parameters in the ADNI database, and all participants were included in the ADNI-3 database. (2) All participants were aged between 60 and 85 years, had completed ≥ 10 years of education, were White, and did not have any neurological disease other than AD. (3) The CN group had a Mini-Mental State Examination (MMSE) score of ≥ 24 and a Clinical Dementia Rating Sum of Boxes (CDR-SB) score of 0. (4) The AD group fulfilled the National Institute of Neurological and Communicative Disorders and Stroke and Alzheimer’s Disease and Related Disorders Association criteria for probable AD, with an MMSE score of < 24and a CDR-SB score of ≥ 1.0. (5) Participants were matched for age, sex, ethnicity, handedness, number of education years, and cognitive function MMSE score between the CN and AD groups and across MRI scanners using three 3-Tesla MRI scanners, namely, Prisma Fit (Siemens Healthineers, Erlangen, Germany) and Signa HDxt and Discovery MR750 (GE Healthcare, Milwaukee, Wis, United States), each with a different protocol. In addition, the ALPS index was higher in women than in men [26]. Our cohort consisted of < 30% women; thus, we decided to include only men to minimize the effect of sex on the ALPS index.

Consequently, the present study included 45 patients with AD and 82 CN participants with clinical neuropsychological scores and dMRI data from the ADNI-3 database. The dMRI data were obtained using three 3-Tesla MRI scanners—Prisma Fit, Signa HDxt, and Discovery MR750—each with a different protocol. Table 1 shows the demographic and clinical data of the study participants.

**Table 1:** Demographic characteristics of participants

	Discovery MR750		Signa HDxt		Prisma fit	
	CN	AD	CN	AD	CN	AD
*N*	29	25	14	11	39	9
Age, years	73.7 ± 7.5	75.4 ± 6.9	74.9 ± 7.8	75.0 ± 6	74.2 ± 7.2	74.0 ± 5.5
Sex, male %	100.0					
Education, years	17.4 ± 2.4	15.6 ± 3.0	17.4 ± 2.8	16.7 ± 2.8	17.0 ± 2.3	15.7 ± 1.8
Ethnicity	White					
Handedness	Right					
MMSE	28.2 ± 2.9	23.4 ± 1.9	28.8 ± 1.4	23.4 ± 2.1	28.3 ± 1.7	23.5 ± 1.5
MoCA	24.5 ± 4.0	18.0 ± 3.9	24.8 ± 2.7	16.9 ± 5.1	24.7 ± 3.2	16.7 ± 3.1
CDR-SB	0.0 ± 0.0	4.7 ± 1.4	0.0 ± 0.0	4.1 ± 1.4	0.0 ± 0.0	4.6 ± 1.9
ADAS 11	7.5 ± 7.3	19.3 ± 7.2	6.5 ± 3.6	22.4 ± 6.1	7.3 ± 5	17.9 ± 5.5
ADAS 13	11.9 ± 9.4	29.5 ± 8.4	10.7 ± 5.7	32.5 ± 7.5	11.8 ± 8	28.4 ± 7.3
ADAS Q4	3.8 ± 2.3	8.7 ± 1.3	4.1 ± 2.6	8.4 ± 1.1	3.9 ± 2.7	8.7 ± 1.1
RAVLT-immediate	42.0 ± 9.7	21.0 ± 5.8	40.6 ± 12.6	19.3 ± 6.2	41.0 ± 10.1	20.2 ± 4.6
RAVLT-learning	5.0 ± 2.5	2.0 ± 1.6	4.1 ± 2.3	1.8 ± 2.4	6.3 ± 2.5	2.5 ± 1.4
RAVLT-forgetting	4.0 ± 1.9	4.1 ± 1.8	3.8 ± 1.9	3.9 ± 1.6	4.6 ± 2.8	4.3 ± 1.8
RAVLT-%-forgetting	42.3 ± 26.3	84.9 ± 25.9	46.5 ± 25.6	92.7 ± 11.9	45.4 ± 29.1	86.7 ± 29.8
Logical Memory	12.5 ± 4	1.7 ± 1.9	11.0 ± 2.6	2.7 ± 2.2	12.0 ± 4.6	1.5 ± 2.5
TMT-B	91.7 ± 63.0	194.7 ± 94.6	75.7 ± 23.7	224.3 ± 93.2	89.5 ± 47.7	136.2 ± 44.7
Ecog-Pt	1.4 ± 0.5	2.1 ± 0.6	1.3 ± 0.3	2.0 ± 0.7	1.4 ± 0.3	2.2 ± 0.6
Ecog-SP	1.4 ± 0.6	2.8 ± 0.5	1.3 ± 0.4	2.7 ± 0.7	1.3 ± 0.4	2.5 ± 0.4
FAQ	1.3 ± 4.7	13.8 ± 5.6	0.6 ± 1.5	15.7 ± 7.7	1.0 ± 3.0	12.8 ± 6.3
FDG-PET SUVRs	–	1.13 ± 0.11	–	1.10 ± 0.18	–	1.14 ± 0.10
AV45-PET SUVRs	–	1.36 ± 0.25	–	1.45 ± 0.16	–	1.49 ± 0.26

*MMSE* Mini-Mental State Examination, *MoCA* Montreal Cognitive Assessment, *CDR-SB* Clinical Dementia Rating Sum of Boxes, *ADAS* Alzheimer’s Disease Assessment Scale, *TMT-B* Trail Making Test Part B, *ECog* Everyday Cognition, *FAQ* Functional Activities Questionnaire, *FDG* ^18^F-fluorodeoxyglucose, *AV45* ^18^F-florbetapir

### Neuropsychological assessments

The following neuropsychological tests were administered in this study: MMSE [27]; Montreal Cognitive Assessment (MoCA) [28], which is a brief questionnaire that measures global cognitive impairment; CDR-SB [29], which classifies patients’ cognitive statuses over six domains of cognitive and functional performance; Alzheimer’s Disease Assessment Scale (ADAS) [30], which includes 28 tasks (11 tasks in ADAS 11 for assessing the memory, language, and praxis domains; 13 tasks in ADAS 13 that include additional tests of delayed word recall and a number cancelation or maze tasks; and 4 tasks in ADAS Q4 for assessing word recognition); Rey Auditory Verbal Learning Test (RAVLT) [31], which is widely used to evaluate anterograde verbal episodic memory in patients; Trail Making Test Part B (TMT-B) [32]; which is an indicator of visual scanning, graphomotor speed, and executive function; Everyday Cognition (ECog) [33], which can be used as part of Alzheimer's disease diagnosis to measure cognitive decline in different areas, including the study partner reported version (ECogSP), and the patient reported version (ECogPT); logical memory delayed recall total (LDELTOTAL) [34], which measures episodic memory; time to complete part B of the Trail Making Test score (TRABSCOR) [35], which measures cognitive flexibility; and the Functional Activities Questionnaire (FAQ) [36], which rates patients’ ability to independently complete activities of daily living.

### Other biomarkers

Two types of SUVRs were obtained from the ADNI database, including FDG-PET SUVRs, which serve as markers of glucose metabolism, and AV45-PET SUVRs, which serve as markers of amyloid deposit. Additionally, FDG-PET and AV45-PET SUVRs were calculated for each participant at ADNI core [37] laboratories following a standardized pipeline (http://adni.loni.usc.edu/methods/pet-analysis/). FDG-PET SUVRs were calculated as the mean uptake of the left and right angular, bilateral posterior cingulate cortex, and inferior temporal gyri normalized by the uptake of the pons/cerebellar vermis region. The AV45-PET SUVRs were calculated as the average of the uptake values of the frontal, angular/posterior cingulate, lateral parietal, and temporal cortices divided by the mean uptake values of the cerebellum.

### MRI acquisition

dMRI data were acquired for each participant using either a 3-Tesla Siemens Prisma Fit scanner or GE Signa HDxt and Discovery MR750 scanners with the acquisition parameters shown in Table 2. Additional imaging details are provided at http://adni.loni.usc.edu/methods/documents/mri-protocols/.

**Table 2:** Diffusion magnetic resonance imaging acquisition parameters

Vendor	GE		Siemens
Model	DISCOVERY MR750	Signa HDxt	Prisma Fit
Repetition time [ms]	9050	13,000	7200
Echo time [ms]	63.0	68.5	56.0
Flip angle [deg]	90	90	90
Field of view [mm]	350 × 350 × 159	350 × 350 × 159	232 × 232 × 160
Matrix size	128 × 128	128 × 128	116 × 116
No. of slices (axial)	46	46	55
Voxel size [mm]	1.37 × 1.37 × 2.70	1.37 × 1.37 × 2.70	2.00 × 2.00 × 2.00
Bandwidth [Hz/Px]	1953	1953	2270
Echo spacing [ms]	0.556	0.568	0.580
*b*-values [s/mm^2^]	0/1000	0/1000	0/1000
No. of directions for *b* = 0/1000 s/mm^2^	5/41	5/41	7/48

Each dMRI examination was performed using three 3-T MRI scanners: Siemens Prisma Fit, GE Signa HDxt, and GE Discovery MR750

In brief, the following parameters were used for Prisma Fit: *b* = 0/1000 s/mm^2^, non-diffusion-weighted image = 7, 48 MPG axes, repetition time (TR) = 7200 ms, TE = 56.0, flip angle of 90°, 2.00 mm isotropic voxel, and 55 axial slices. Signa HDxt had the following parameters: *b* = 0/1000 s/mm^2^, non-diffusion-weighted image = 5, 41 MPG axes, TR = 13,000 ms, TE = 68.5 ms, flip angle of 90°, 1.37 mm $\times$ 1.37 mm voxel, 2.70 slice thickness, and 46 axial slices. DISCOVERY MR750 had the following parameters: *b* = 0/1000 s/mm^2^, non-diffusion-weighted image = 5, 41 MPG axes, TR = 9050 ms, TE = 63.0 ms, flip angle of 90°, 1.37 mm $\times$ 1.37 mm voxel, 2.70 slice thickness, and 46 axial slices.

### dMRI processing

The acquired dMRI data were pre-processed using FSL 6.0.1 and MRTrix3, following the protocol used in recent studies on optimal pre-processing [38]. First, MR magnitude images were denoised using Marchenko–Pastur principal component analysis (MP-PCA) as a denoising algorithm [39] and corrected for Gibbs artifacts [40]. Next, an analytical approach was adopted, and the noise standard deviation estimated by applying MP-PCA was used to reduce possible biases caused by a Rician noise distribution. Additionally, the effect of eddy currents and motion, including dMR images, and B1 inhomogeneity were corrected using the “*eddy*” commands in the FSL software [41]. Subsequently, the resulting dMR images were fitted to the DTI model by estimating with ordinary least squares to generate FA and diffusion coefficient maps in the direction of the x-axis (right–left; Dxx), y-axis (anterior–posterior; Dyy), and z-axis (inferior–superior; Dzz) using the “*dtifit*” command in the FSL software [42]. The diffusion coefficient and FA maps were finally assessed to determine whether the data were free from severe artifacts, such as gross geometric distortion, signal dropout, and bulk motion.

### ALPS index calculation

**Fig. 1:**
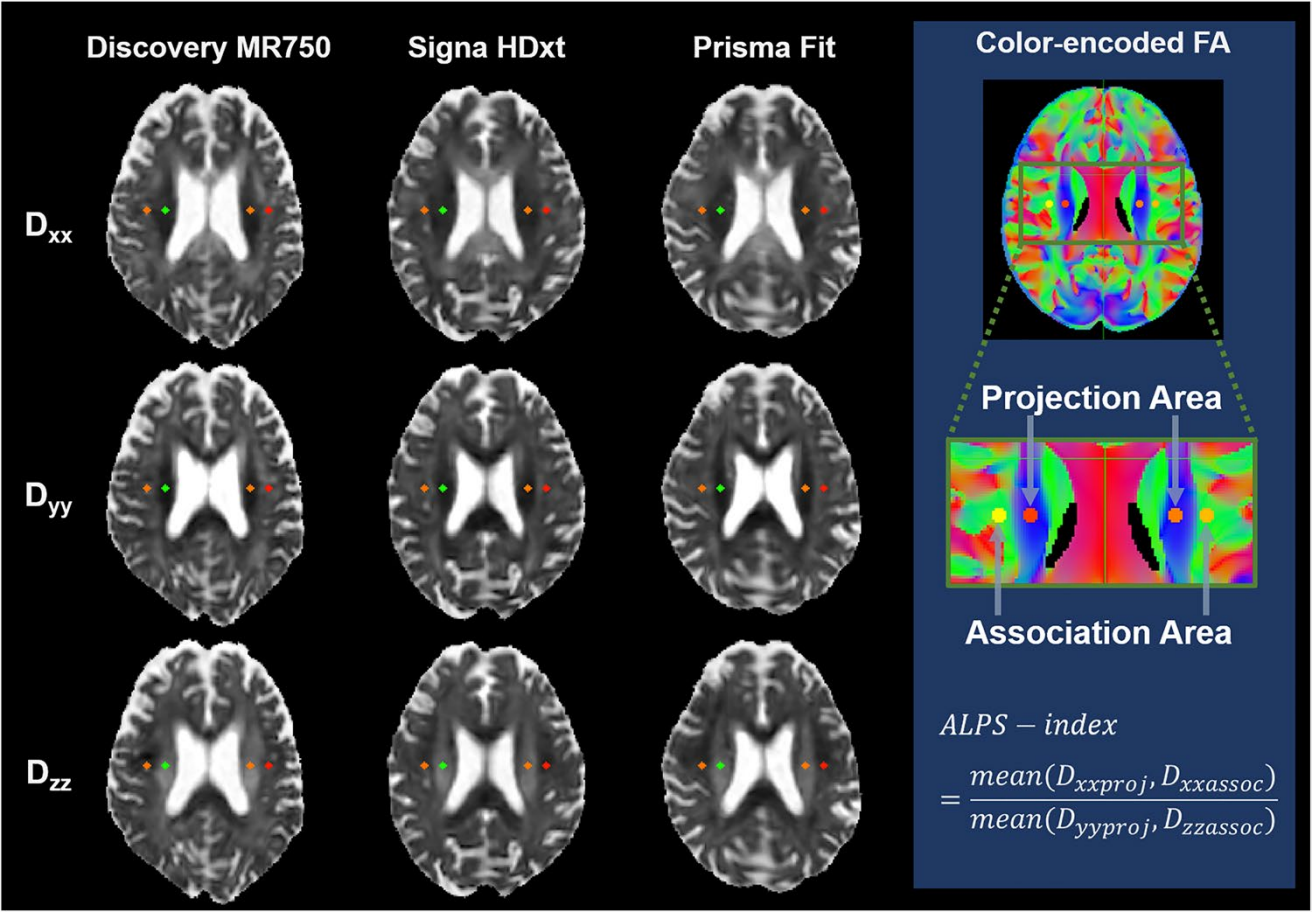
Diffusivity map of each scanner and ALPS index calculation. The ALPS index is a ratio of the mean x-axis diffusivity in the projection area (${D}_{xxproj}$) and x-axis diffusivity in the association area (${D}_{xxassoc}$) to the mean y-axis diffusivity in the projection area (${D}_{yyproj}$) and the z-axis diffusivity in the association area (${D}_{zzproj}$). An ALPS index close to 1.0 reflects minimal diffusivity along the perivascular space, whereas a higher value indicates greater diffusivity

The ALPS index based on DTI-ALPS was calculated according to a method proposed by Taoka et al. [5]. First, the FA maps of all participants were registered linearly and then nonlinearly into the high-resolution FMRIB58_FA standard-space image. Second, one subject with the smallest degree of warping (i.e., with the smallest sum of squared differences) was selected for the region-of-interest (ROI) placement. Third, using this subject’s native color-coded FA map, 5 × 5 mm^2^ ROIs were placed in the projection and association areas at the level of the lateral ventricle bodies in the left and right hemispheres. Dominant fibers run in the z-axis direction, perpendicular to both the x- and y-axes, in the projection area, whereas they run in the y-axis direction, perpendicular to both the x- and z-axes, in the association area. Fourth, the resulting ROIs were then registered to the same FA template. The ROI position was manually checked for each participant. Manual corrections were not performed because all ROIs were correctly placed. Finally, as shown in Fig. 1, the left and right ALPS index was calculated as follows:

1 $$\begin{array}{c}ALPS-index=\frac{mean\left({D}_{xxproj},{D}_{xxassoc}\right)}{mean\left({D}_{yyproj},{D}_{zzassoc}\right)},\end{array}$$

which is the ratio of the mean x-axis diffusivity in the projection area (${D}_{xxproj}$) and x-axis diffusivity in the association area (${D}_{xxassoc}$) to the mean y-axis diffusivity in the projection area (${D}_{yyproj}$) and the z-axis diffusivity in the association area (${D}_{zzproj}$). An ALPS index close to 1.0 indicates minimal diffusivity along the perivascular space, whereas a higher value indicates greater diffusivity.

### COMBAT harmonization methods

In this study, $y(i, j, v)$ was the $v$ th anatomical variable, such as the left and right ALPS indexes, for MRI scanner $i$ and the $j$ th subject. The COMBAT harmonization method in this study was implemented using MATLAB (R2019a) and is described below.

COMBAT is a GLM extension with an empirical Bayesian-based harmonization method that uses covariate regression for data harmonization. The COMBAT harmonization method has been recently introduced in neuroimaging, although it was originally used as a batch effect correction tool in genomics [25]. For instance, Fortine et al. harmonized the cortical thickness from multisite structural MRI [43] and FA and MD from multisite DTI data [21] using COMBAT to improve the statistical power. The COMBAT formula is as follows:

2 $$\begin{array}{c}y\left(i,j,v\right)=a\left(v\right)+{X}^{T}\left(i,j\right)\beta \left(v\right)+\gamma \left(i,v\right)+\delta \left(i,v\right)\epsilon \left(i,j,v\right),\end{array}$$

where $a\left(v\right)$ is the average diffusion metric at the reference site within the $v$ th anatomical variable; $\beta \left(v\right)$ is the $p\times 1$ vector of coefficients associated with the design matrix of biological covariates, including age, sex, and subject type (i.e., AD or CN); $X(i,j)$ is the design matrix of the $v$ th anatomical variable; $p$ is the number of biological covariates; and $\epsilon (i,j,v)$ is the error term and follows a normal distribution with a mean of zero and a variance of ${\sigma }^{2}(v)$. The terms $\gamma \left(i,v\right)$ and $\delta \left(i,v\right)$ are the additive and multiplicative scanner effects on the $v$ th anatomical variable. The terms ${\gamma }^{*}\left(i,v\right)$ and ${\delta }^{*}(i,v)$ were estimated using an empirical Bayesian framework in COMBAT harmonization. The COMBAT harmonized values, ${y}^{COMBAT}(i,j,v)$, can be described as follows:

3 $$\begin{array}{c}{y}^{COMBAT}\left(i,j,v\right)=\frac{y\left(i,j,v\right)-\widehat{a}\left(v\right)-{X}^{T}\left(i,j\right)\widehat{\beta }\left(v\right)-{\gamma }^{*}\left(i,v\right)}{{\delta }^{*}\left(i,v\right)}\widehat{a}\left(v\right)+{X}^{T}\left(i,j\right)\widehat{\beta }\left(v\right),\end{array}$$

where $\widehat{\beta }\left(v\right)$ and $\widehat{a}\left(v\right)$ represent estimated coefficients associated with the biological covariates and estimated population mean of the $v$ th anatomical variable, respectively.

### Statistical analysis

The Cohen’s *d* effect size of the ALPS index between groups (i.e., AD and CN) and scanners (i.e., Siemens Prisma Fit and GE Signa HDxt and Discovery MR750) was calculated to evaluate harmonization performance. Cohen’s *d* formula is as follows:

4 $$\begin{array}{c}{S}_{c}=\sqrt{\frac{{n}_{1}{s}_{1}^{2}+{n}_{2}{s}_{2}^{2}}{{n}_{1}+{n}_{2}}},\end{array}$$

5 $$\begin{array}{c}Cohe{n}^{^{\prime}}s d=\frac{\left|{\overline{x} }_{1}-{\overline{x} }_{2}\right|}{{S}_{c}},\end{array}$$

where ${n}_{1}$ and ${n}_{2}$ represent the numbers of subjects in populations 1 and 2, respectively. ${\overline{x} }_{1}$ and ${\overline{x} }_{2}$ and ${s}_{1}$ and ${s}_{2}$ represent the average and standard deviation of each variable in populations AD and CN, respectively. This study calculated Cohen’s *d* for the ALPS index between all site, model, and protocol combinations according to each scanner effect. Cohen’s *d* approaches a smaller value as the scanner effect decreases and must be equal to 0 if there is no scanner effect. Additionally, to assess the effect of scanner variations on the ALPS index before and after harmonization, which is independent of age, sex, and group (i.e., AD and CN), GLM was used for analysis of variance (ANOVA), with the ALPS index as the independent variable and scanner, age, and group as the dependent variables. Sex was not included as a factor because this study included only men.

Moreover, Welch’s *t* test was performed to compare the difference in ALPS indexes between AD and CN groups before and after harmonization. Pearson’s correlation coefficient was calculated to assess the relationships between the ALPS index and cognitive score, FDG-PET, and AV45-PET SUVRs for all participants. A *P* value of < 0.05 was considered statistically significant.

## Results

### Evaluation of harmonization performance

**Fig. 2:**
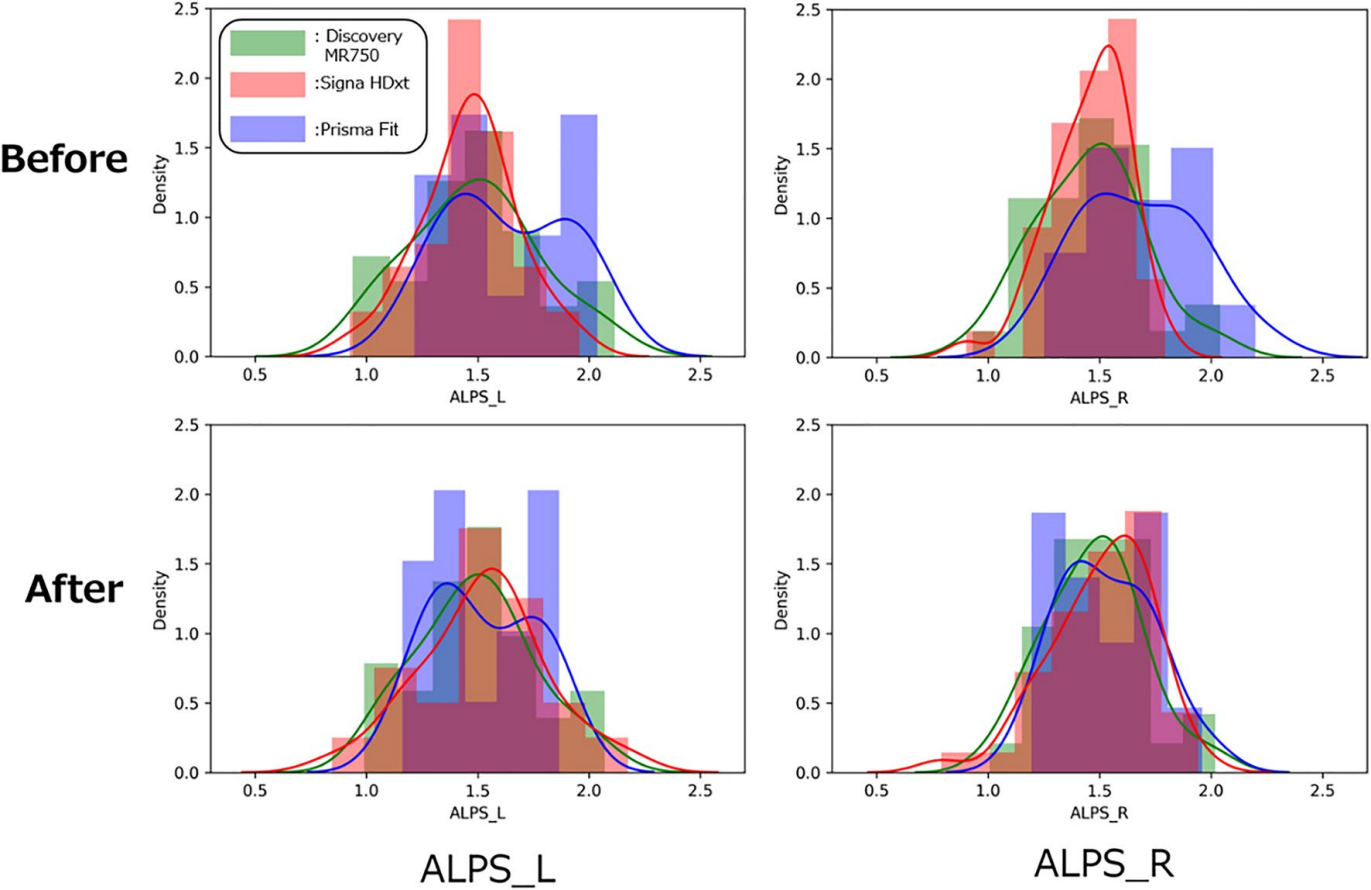
ALPS index distribution in CN before and after harmonization. The ALPS index distribution was closer after harmonization. A solid curve indicates kernel density estimation. Red, Prisma Fit; blue, Signa HDxt; red, Discovery MR750

Table 3 shows the ALPS indexes in AD and CN before and after harmonization. Additionally, Fig. 2 shows the distribution of the ALPS indexes in CN before and after harmonization. The distributions of the ALPS indexes on each scanner were not matched before harmonization, although we matched the participants for age, sex, ethnicity, handedness, number of education years, and cognitive function (MMSE and MoCA) across MRI scanners to reduce the sampling bias and focus on measurement bias, such as the effect of scanner variations on the ALPS index. The ALPS index was the largest for Prisma Fit, followed by Discovery MR750 and Signa HDxt before COMBAT harmonization (Table 3). The effect size (i.e., Cohen’s *d*) of the scanner in the ALPS index decreased after harmonization (Table 4). Before harmonization, the differences in Cohen’s *d* in the ALPS indexes among the scanners were ≤ 0.948. After harmonization, the ALPS index distribution was closer among scanners, with a difference in Cohen’s *d* of ≤ 0.183. Although the Cohen’s *d* between Discovery MR750 and Prisma Fit was relatively small even before harmonization, the Cohen’s *d* decreased after harmonization.

**Table 3:** Mean ALPS index before and after harmonization

Group	Scanner	Left ALPS index		Right ALPS index	
		Before	After	Before	After
CN	Discovery MR750	1.55 ± 0.25	1.55 ± 0.22	1.51 ± 0.21	1.52 ± 0.19
	Signa HDxt	1.50 ± 0.18	1.56 ± 0.24	1.48 ± 0.15	1.54 ± 0.19
	Prisma Fit	1.63 ± 0.27	1.52 ± 0.23	1.68 ± 0.26	1.53 ± 0.21
AD	Discovery MR750	1.50 ± 0.32	1.50 ± 0.28	1.43 ± 0.22	1.45 ± 0.20
	Signa HDxt	1.34 ± 0.14	1.37 ± 0.19	1.37 ± 0.15	1.41 ± 0.20
	Prisma fit	1.49 ± 0.19	1.39 ± 0.17	1.56 ± 0.28	1.43 ± 0.22

**Table 4:** Effect size of scanner differences in the ALPS index before and after harmonization

Model	Left ALPS index		Right ALPS index	
	Before	After	Before	After
Discovery MR750 vs. Signa HDxt	0.747	0.183	0.336	0.033
Discovery MR750 vs. Prisma Fit	0.140	0.122	0.237	0.121
Signa HDxt vs. Prisma Fit	0.948	0.057	0.603	0.095

The effect size of scanner differences in the ALPS index is shown as Cohen’s *d*

**Fig. 3:**
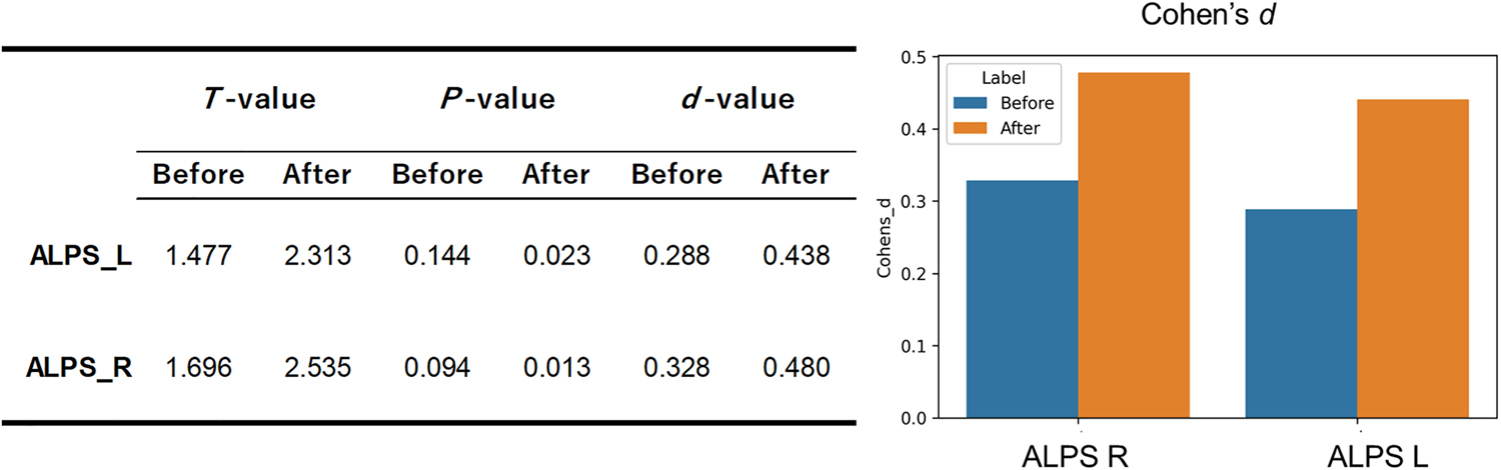
Group difference between AD and CN. The group differences of the left and right ALPS indexes between CN and AD are shown before (blue bar) and after (red bar) COMBAT harmonization. COMBAT harmonization removed the scanner differences from multiscanner data and arranged the ALPS index distribution. Consequently, the group difference after harmonization was 1.5 times as the Cohen’s *d* and the *P* value decreased. This indicates that the statistical power (i.e., 1-$\beta$) improved. Thus, COMBAT increased the statistical power of the multisite ALPS index between CN and AD by almost double

Table 5 presents the results of the scanner effect analysis with respect to the comparison between AD and CN groups using GLM. The *F* value and partial ${\eta }^{2}$ of the scanner largely decreased in both the left and right ALPS indexes and showed no significant difference in the ANOVA test of the scanner effect after harmonization, even though there was a significant difference before harmonization. Consequently, Cohen’s *d* of the left and right ALPS indexes between AD and CN increased from 0.288 to 0.438 and from 0.328 to 0.480, respectively, approximately doubling the group difference between AD and CN (Fig. 3). Furthermore, the ALPS index between AD and CN was significantly different after harmonization (*P* < 0.05) but not before it (left ALPS index, *P* = 0.144; right ALPS index, *P* = 0.094).

**Table 5:** GLM analysis of the scanner effect in the group comparison between AD and CN

	Left ALPS index		Right ALPS index	
	Before	After	Before	After
*F* value	3.28	0.37	7.38	0.12
*P* value	< 0.05	0.69	< 0.001	0.88
Partial *η*^*2*^	0.05	0.01	0.11	0.00

### Correlation between the ALPS index and cognitive function

Pearson’s correlation coefficient was calculated to assess the relationships between the ALPS index and cognitive score, FDG-PET SUVRs, and AV45-PET SUVRs (Table 6). The ALPS index was positively correlated with MMSE, MoCA, RAVLT-immediate, RAVLT-learning, logical memory, and FDG-PET SUVRs; higher cognitive scores indicated better cognitive function and higher FDG-PET SUVRs indicated better glucose metabolism. Conversely, the ALPS index was negatively correlated with ADAS 11, ADAS 13, ADAS Q4, RAVLT-%-forgetting, TMT-B, Ecog-Pt, Ecog-SP, CDR-SB, FAQ, and AV45-PET; higher cognitive scores indicated worse cognitive function and higher AV45-PET SUVRs indicated high A $\beta$ deposition. Thus, the ALPS index was negatively correlated with cognitive function and A $\beta$ deposition and was positively correlated with glucose metabolism. Moreover, Pearson’s correlation coefficients were larger after harmonization, except for FDG-PET SUVRs.

**Table 6:**
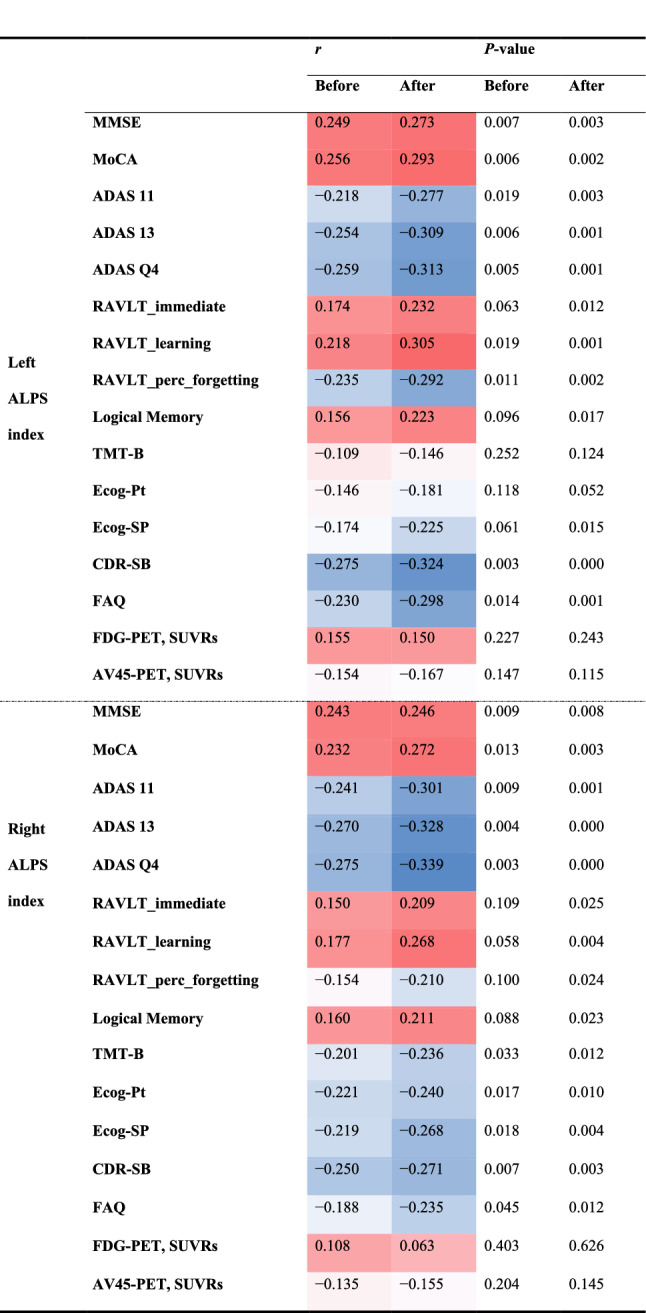
Correlation between the ALPS index and cognitive scores

COMBAT harmonization increased the correlation between the ALPS index and cognitive function by reducing the variation caused by scanner differences. Red cells indicate a positive correlation and blue cells indicate a negative correlation

*MMSE* Mini-Mental State Examination, *MoCA* Montreal Cognitive Assessment, *CDR-SB* Clinical Dementia Rating Sum of Boxes, ADAS Alzheimer’s Disease Assessment Scale, *TMT-B* Trail Making Test Part B, *ECog* Everyday Cognition, *FAQ* Functional Activities Questionnaire, *FDG-PET* [^18^F] fluorodeoxyglucose-positron emission tomography, AV45 [^18^F] florbetapir, *SUVRs* standardized uptake value ratios

## Discussion

This study evaluated the performance of COMBAT to statistically harmonize site variations in the ALPS index caused by differences in scanners, sites, and protocols and assessed the ability of COMBAT harmonization to improve the correlations between the ALPS index and cognitive score, [^18^F]-FDG-PET SUVRs, and [^18^F]- AV45-PET SUVRs. Our results demonstrated that COMBAT harmonized the effect of scanner differences and increased Cohen’s *d* of the ALPS index between AD and CN by almost double. Moreover, Pearson’s correlation between the ALPS index and neurocognitive score coefficients was larger after harmonization. Kamagata et al. [15] reported a significantly lower ALPS index in AD than in CN and correlations with cognitive functions, such as the MMSE, CDR-SB, FAQ, ADAS-11, and ADAS-13. Our study revealed a significantly lower ALPS index in AD and a stronger correlation with the cognitive score, which was consistent with the findings reported by Kamagata et al. [15].

Taoka et al. [16] described how the ALPS index is influenced by MR acquisition parameters. First, they showed that the ALPS index changed by approximately 0.10 in the scan–rescan dMRI. Our study demonstrated that COMBAT harmonized the multisite ALPS indexes and reduced the change among scanners from 0.14 to 0.20 to 0.02 up to the scan–rescan level (Table 3). Second, Taoka et al. [16] found that the ALPS index was influenced by the number of MPG axes and TE in the imaging protocol and MRI scanner. Literature reports have described an increased ALPS index of up to 0.2 as the number of MPG axes changed from 12 to 30 axes, a decreased ALPS index by up to 0.2 as the TE changed from 65 to 100 ms, and an increased ALPS index by up to 0.1 due to MRI scanner differences [16]. Therefore, the ALPS index might increase as the number of MPG axes increases or the TE decreases. Our study showed that the ALPS index was the largest for Prisma Fit (MPG axes, 48; TE, 56.0 ms), followed by Discovery MR750 (MPG axes, 41; TE, 63.0 ms) and Signa HDxt (MPG axes, 41; TE, 68.5 ms) before COMBAT harmonization. We also found that the ALPS index of Prisma Fit with a shorter TE and higher MPG direction was higher than that of other scanners (Table 2). This tendency of the ALPS index to change depended on the dMRI acquisition parameters, which is consistent with the findings of a previous study [16].

COMBAT harmonization reduced scanner differences in the multiscanner dMRI data, minimized interscanner ALPS index distributions, and eliminated ALPS index variations among MR scanners. Consequently, after harmonization, the group difference between AD and CN (Cohen’s *d*) was almost doubled and was significant after harmonization but not before harmonization (Fig. 3). The statistical power (i.e., 1-$\beta$) improved from 0.32 to 0.65 in the left ALPS index and from 0.40 to 0.73 in the right ALPS index because the significance level was 0.05 and the sample comprised only AD and CN subjects in this study. Thus, COMBAT increased the statistical power of the multisite ALPS index between CN and AD by approximately twofold. The sample size needs to be corrected by at least 454 (151 AD and 303 CN subjects) if the statistical power (i.e., 1-$\beta$) is required to be 0.80 to detect a significant difference in the ALPS index, considering that the significance level was 0.05 and Cohen’s *d* of the ALPS index was 0.288–0.328 before harmonization, as shown in this study. Conversely, the sample size needs to be corrected by at least 188 (63 AD and 125 CN subjects) if the statistical power (i.e., 1-$\beta$) is required to be 0.80, considering that the significance level was 0.05 and Cohen’s *d* of ALPS index was 0.438–0.480 after harmonization in this study. Thus, COMBAT could also help reduce the cost of data correction in multisite studies using the ALPS index to detect changes in the glymphatic system associated with pathology.

The ALPS index has been reported to reflect the function of the glymphatic system and was associated with cognitive function and A $\beta$ deposition based on AV45-PET in AD [15]. Our study showed that COMBAT harmonization increased the correlation between the ALPS index and cognitive function and A $\beta$ deposition based on AV45-PET by reducing the variation caused by site, scanner, and protocol differences, even though cognitive scores and AV45-PET SUVRs were not included in the COMBAT model. The ALPS index was positively correlated with MMSE, MoCA, RAVLT-immediate, RAVLT-learning, logical memory, and FDG-PET SUVRs, indicating that higher cognitive scores imply better cognitive function and that higher FDG-PET SUVRs imply better glucose metabolism. Conversely, the ALPS index was negatively correlated with ADAS 11, ADAS 13, ADAS Q4, RAVLT-%-forgetting, TMT-B, Ecog-Pt, Ecog-SP, CDR-SB, FAQ, and AV45-PET, indicating that higher cognitive scores imply worse cognitive function and higher AV45-PET SUVRs imply high A $\beta$ deposition. Thus, the ALPS index was negatively correlated with cognitive function and A $\beta$ deposition and positively correlated with glucose metabolism. Moreover, Pearson’s correlation coefficients were larger after harmonization, except for FDG-PET SUVRs. These results were consistent with the findings of a previous study [15]. This suggests that COMBAT could harmonize the ALPS index calculated from multisite dMRI while retaining and clarifying the relationship between the ALPS index and cognitive function and Aβ deposition based on AV45-PET.

This study had some limitations. First, this study was not validated using traveling-subject (TS) data that include only measurement bias, such as scanner and protocol effects, and not sampling bias, such as age, sex, and education effects. TS data are required to confirm whether COMBAT can harmonize measurement bias. However, traveling to some sites to obtain brain dMRI is challenging for patients, such as those with AD. Second, this study excluded scan–rescan data to compare the differences in effects on the ALPS index between scan–rescan and site-specific differences. Third, this study included only men. Thus, further studies including both women and men are needed. Previous reports on harmonization for cortical thickness [43] and DTI metrics [21] revealed that COMBAT worked well for data from both men and women to reduce site variations. Thus, COMBAT might be suitable for harmonizing the multisite ALPS index even with data from women and men. Fourth, this study investigated the effects of differences in scanner models on the ALPS index; however, because of data deficiency, it did not investigate the effect of intrascanner differences on the ALPS index. Additional studies are required to confirm the effect of individual scanner differences on the ALPS index using TS data. Finally, at present, there is no agreement on a gold standard or ground truth for pre-processing of the ALPS index based on DTI-ALPS. However, differences in image pre-processing affect reproducibility [44]. Therefore, we applied common pre-processing techniques, such as MP-PCA denoising, correction for Gibbs artifacts, and evaluation of the effect of eddy current and head motion on dMRI data, in this study [15, 16, 45]. Further studies are needed to investigate the effect of pre-processing differences on the ALPS index.

Our study revealed that COMBAT harmonization was useful for eliminating variations in the ALPS index (calculated from DTI-ALPS using dMRI) caused by scanner, site, and protocol differences. The study also confirmed and clarified the relationships between the ALPS index and cognitive function and A $\beta$ deposition based on AV45-PET. Thus, our findings indicate that a multisite study in a large cohort could correctly detect changes in the glymphatic system caused by pathological changes after harmonizing the ALPS index using COMBAT and facilitate the use of the ALPS index in multi-center studies.
